# Methodology and Design for Abrasive Tools in Precision Grinding Processes

**DOI:** 10.3390/ma19132913

**Published:** 2026-07-07

**Authors:** Wojciech Kacalak, Katarzyna Tandecka, Łukasz Rypina, Filip Szafraniec, Thomas G. Mathia

**Affiliations:** 1 Department of Engineering and Informatics Systems, Faculty of Mechanical Engineering and Energy, Koszalin University of Technology, 75-620 Koszalin, Poland; wojciech.kacalak@tu.koszalin.pl (W.K.); lukasz.rypina@tu.koszalin.pl (Ł.R.); filip.szafraniec@tu.koszalin.pl (F.S.); 2 Laboratoire de Tribologie et Dynamique des Systemes (LTDS), Ecole Centrale de Lyon, Centre National de la Recherche Scientifique, 69134 Lyon, France

**Keywords:** abrasive tools, aggregate structures, grinding wheel topography, active grain distribution, *Shos* parameter, probabilistic modeling, FEM simulation, tool wear, grinding efficiency

## Abstract

**Highlights:**

- *Shos* factor was used for the assessment of active vertices of grains and the wear of the grinding wheel.
- Aggregated abrasives created a wider area of cutting zone than that created by a single abrasive grain.
- The geometry of the aggregated abrasive helped reduce lateral pile-up and improve chip formation.
- Aggregate-based abrasive tools may support more stable and precise grinding under appropriately selected process conditions.

**Abstract:**

The workability of abrasive tools for precision grinding is significantly affected by the quality of active surface of grinding wheel in terms of the number, form, and sharpness of active abrasive grains. The aim of this study is to introduce a method for optimizing the abrasive tools based on active surface topography analysis, tool wear diagnostics, and numerical simulation of micro-cutting by one active grain and abrasive aggregate. Particular attention is given to the *Shos* parameter, which characterizes the machining potential of a grinding wheel by combining information on the height and sharpness of active abrasive grain vertices. Changes in this parameter allow observation of the blunting and wear of the active surface. Numerical simulation shows that material removal by one active grain and by an abrasive aggregate differs significantly. According to the obtained data, under the assumed micro-cutting conditions, the aggregate geometry reduced lateral material displacement and promoted chip formation. The coefficient of material removal efficiency for aggregate was equal to kr = 0.93 compared to kr = 0.37 for one grain. Therefore, abrasive aggregates have an effect on the material removal process and may support the improvement of the stability of precision grinding. Thus, it can be concluded that further improvements in abrasive tools require considering controlled active surface structures and abrasive aggregates, as well as diagnostic parameters that relate tool topography to wear and machining efficiency.

## 1. Introduction

Grinding and abrasive finishing continue to be crucial for precision machining applications in which the use of difficult-to-machine materials demands high dimensional accuracy, surface integrity control, and functional stability of parts. Material removal in grinding is caused by a combination of complicated processes involving interactions between abrasive grains, workpiece material, thermodynamics, and wear of tools [1,2]. Therefore, abrasive tool development cannot be reduced to the selection of nominal grain size, bond type, or general process parameters; it requires taking into account the local state of active grain vertices. Only a fraction of abrasive grains in abrasive machining are active simultaneously, and their condition determines the actual contact conditions and material removal mechanism [3]. Conventional grinding wheels feature stochastic distribution of abrasive grains, thus making control of the number, protrusion, and geometry of active cutting edges impossible [4,5]. Such phenomena are typical for abrasive-film finishing, where local pressure distribution and tool compliance have a significant effect on the effective contact area and material removal [6].

Recent developments in grinding-tool design reveal a trend towards creating porous and structured abrasive systems. Triply periodic minimal surface grinding wheels and liquid body armor grinding wheels are examples of recent proposals for improving the coolant flow, chip evacuation, and thermal behavior of grinding zones [7,8]. Discontinuous abrasive films are another method of reducing the risk of deep scratch formation in finishing through local interruptions in active surface [9]. At the same time, advanced lubrication techniques and chemical–mechanical methods are applied for reducing thermal load and ensuring better surface integrity during machining of hard-to-cut and brittle materials [10,11]. Nonetheless, the morphology of micro-chips created by abrasive finishing reveals that the material separation process continues to be very local and highly sensitive to grain geometry [12]. In the grinding of titanium alloys and nickel-based superalloys, these problems become particularly relevant due to the high susceptibility of the surface layer to mechanical and thermal loads. Residual stresses in machining Ti-6Al-4V are generated depending on chip formation, unloading, and cooling stages, thus making microscopic material flow one of the factors responsible for surface integrity [13]. A machinability assessment of Ti-6Al-4V based on energy criteria suggests that machining of the workpiece involves several physical processes that cannot be described using a single parameter [14]. The superfinishing of Ti-6Al-4V with abrasive films implies that final surface quality depends on grain size, contact-point distribution, and possibility of achieving uniform functional surface [15]. Prediction of surface roughness in grinding has recently been extended to surfaces of complex geometry, where the roughness profile depends on kinematic and grain–workpiece interaction [16]. Hard-brittle composite coatings require analysis of transitions among ductile removal, brittle cracking, and chemically assisted softening [17]. Microfinishing of Nimonic 80A demonstrates that the machining capacity of abrasive film depends on grain apex distribution, wear processes, and chip storage conditions [18].

Functional significance of surface topography is not confined to conventional grinding. Process parameters and scanning strategy in laser powder bed fusion have a significant effect on roughness and defect formation on Ti-6Al-4V and stainless steel surfaces [19,20]. Different variants of microabrasive finishing produce various densities of surface protrusions and contact points despite using identical tool types [21,22]. In electrical discharge machining, surface roughness alone does not distinguish functionally distinct crater morphologies [23]. Surface smoothing with abrasive film coated with graphite demonstrates that surface functionalization through carbon texture formation is possible during finishing [24]. Surface engineering research shows that surface topography, porosity, and multiscale texture affect contact area, wettability, tribology, and biological response [25,26]. The creation of ultrathin carbon textures in abrasive-film finishing proves that surface modification reduces roughness while changing surface chemistry and tribological properties [27]. Textured titanium surfaces and boundary-lubricated textured surfaces confirm that surface features influence lubricant retention and tribological characteristics [28,29]. Thus, in the context of grinding and finishing tools, surface, contact pressure, and texture should be considered a coupled system [30]. Characterization of the surface topography of abrasive tools constitutes another challenge. Classic contact models and roughness parameters constitute a theoretical base for understanding surface behavior, yet they may prove insufficient in analyzing surfaces where only the highest, sharpest grain vertices actively participate in machining [31,32]. Control of the finishing zone and effective contact area in modern dual-roller superfinishing systems relies on roller geometry and compliance [33]. Methods of optical and interferometric surface inspection provide high-resolution surface topography and information about surface features necessary for process development [34,35]. Modern surface metrology has developed from simple profile-based roughness evaluation to areal and informative surface characterization. Conceptual shifts in surface metrology, ISO texture concepts, and information models for surface data contribute to a new understanding of surface topography [36,37,38]. Registration and stitching algorithms are necessary in large-scale or complex topography reconstruction from incomplete measurements [39,40].

Surface topography measurement is influenced significantly by sampling strategy, calibration, and uncertainties. Reviews of sampling strategies and freeform surfaces research emphasize the necessity of considering surface geometry and measurement scale in interpreting areal parameters [41,42]. Calibration studies show that material measures must be chosen carefully in comparing topography-measuring instruments [43]. Coherence scanning interferometry extends noncontact measurement range to surfaces characterized by complex diffraction of reflected light [44]. Analysis of surface topography also includes data processing. Autocorrelation analysis, thresholding, and definition of reference plane greatly affect the interpretation of roughness [45,46,47]. As a result, topographic and topological approaches become increasingly popular as ways of considering surfaces as spatial systems [48,49]. The simulation of rough surfaces provides additional support for linking topography to functionality. Recent developments in surface modeling include coupling selected areal parameters, such as Sdr, Sdq, Spd, and Spc, with functional characteristics of rough surfaces [50]. Simulations of vibratory-polished surfaces prove that measurable microtexture features can be analyzed for predicting service performance [51]. Gaussian process models and transfer learning approaches imply that surface prediction is possible with a minimal amount of experimental data [52,53].

For aperiodic and multiscale surfaces, methods of meshing, bifractal and fractal description are convenient tools for examining contact behavior and distribution of spatial features [54,55]. The concepts of fractal anisotropy and contact mechanics become relevant to abrasive tools, as grain vertices feature irregular shapes and operate on multiple scales [56,57,58]. Analysis of multiscale anisotropy and autocorrelation-based morphology descriptions allow more precise interpretation of complex surface patterns [59,60]. Modeling roughness sequences, roughness power spectra, correlation analysis, and machine learning-based prediction are all helpful when roughness parameters describe the physical surface formation mechanism [61,62,63,64,65]. Methods of signal processing can be used for detecting changes in surface topography and tool condition indicators [66]. In the context of grinding mechanics, simulations of the grinding wheel surface and interactions between abrasive grains and workpiece have been widely used for studying stochastic surface generation [67,68]. Discrete element models prove that both wheel-body structure and surface topography have an effect on the actual grinding contact length [69]. Innovations in grinding-wheel design in the manufacturing of advanced ceramics imply that the structure of the grinding tool determines its efficiency [70].

Studies of surface modification prove that roughness, contact state, and properties of surface layers must be considered together when expecting functional performance [71]. The influence of surface layers on wave propagation and resonance is another example of how surface and subsurface structure may affect the behavior of precision components [72]. Compliant tool studies reveal the role of elastic support and local compliance in controlling finishing processes and preventing excessive loading [73]. For abrasive tools, the most important part of the surface is not the average topography but the set of high, sharp vertices that actively participate in machining. The parameter *Shos* is useful in this context as it combines information about vertex heights and slope sharpness and allows the assessment of machining potential and wear of the active grinding-wheel surface [74,75]. It becomes particularly important in precision grinding, where the number of active grains is very low and initial wear starts from blunting of the highest cutting vertices.

Despite a vast number of studies on grinding mechanics, surface metrology, abrasive-film finishing, structured tools, and rough surface modeling, there is a need for developing a methodology that will combine the assessment of active surface topography, stochastic participation of abrasive grains, tool wear diagnostics, and numerical analysis of material separation. Many works consider either roughness formation, heat generation, texture modeling, or contact mechanics in isolation, whereas few attempt to combine these factors and develop grinding tools with controlled active structure. This work aims to provide a methodology for the improvement of abrasive tools used in precision grinding through combining active surface assessment and numerical simulation of material removal by a single grain and abrasive aggregate. The focus of this work will be put on the condition of the active grains’ vertices, application of the *Shos* parameter for evaluating the machining potential and wear of the active grinding-wheel surface, and dependence of chip formation and lateral pile-up formation on abrasive aggregate geometry. The novelty of this work consists of treating abrasive aggregate as not just an addition to the grinding wheel but as a structural component capable of altering the material removal process through increasing the effective cutting-zone width and restricting lateral material flow. Therefore, the central scientific question of this study is how the controlled structure of the grinding wheel active surface, including abrasive aggregates and sharp active grain vertices, can improve material removal efficiency and reduce lateral pile-up formation in precision grinding.

## 2. Materials and Methods

The methodology adopted in this work combines the analysis of the active surface of abrasive tools with a numerical case study of material removal by a single abrasive grain and by an abrasive aggregate. The first part of the methodology concerns the assessment of the grinding wheel active surface and its machining potential. The second part concerns the analysis of micro-cutting, where the influence of abrasive grain and aggregate geometry on chip formation and lateral pile-up formation was examined.

This approach was adopted because the development of new abrasive tools cannot be based only on general grinding parameters. It is also necessary to take into account the state of active grain vertices, their distribution, the local geometry of cutting edges, and the way in which the workpiece material flows in the cutting zone.

### 2.1. Evaluation of the Active Surface of Abrasive Tools

The active surface of abrasive tools was evaluated with particular attention to the highest and sharpest grain vertices. These vertices are important because they are the first parts of the grinding wheel that come into contact with the workpiece material. In precision grinding, the share of active grains is often small; therefore, the state of these grains has a significant influence on the process.

In the evaluation of the grinding wheel surface, it is important to extract data that are technologically useful and sensitive to changes caused by wear. In the case of ground surfaces, many commonly used parameters describe surfaces where the base of a motif is much larger than its height. In the case of grinding wheel surfaces, the situation is different, because the height of surface motifs is comparable with the dimension of their base. Moreover, the ground surface is shaped in a relatively permanent way, while the grinding wheel active surface changes continuously during the process.

**Figure 1:**
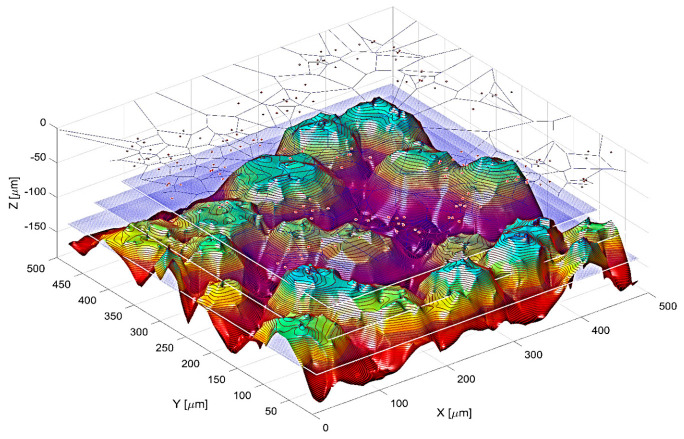
A scheme for the analysis of abrasive grain vertex features located at different levels determined from the highest vertex of the grinding wheel [76].

For this reason, the analysis was focused on the features of grain vertices located above a selected reference plane (Figure 1). Such a plane may be located, for example, at a level from 0.2 to 0.3 of the St parameter value from the highest vertex of the grinding wheel surface.

The use of Voronoi cells may support this analysis. It allows the distances between neighboring grain vertices and the inclination angles of lines connecting these vertices to be determined. For the tested tools, the following parameters were determined: the area of the elevation above the selected plane, the coordinates of elevation vertices, the density of elevations, statistical parameters of vertex positions, and the development of the perimeter of the elevation base.

The useful features of the tool surface include stable renewable sharpness of grain vertices. In precision machining, a large number of micro-corners along a wide cutting front is also beneficial. It is also important that the operating radii of cutting edges are not strongly differentiated.

### 2.2. Shos Parameter and Tool Surface Measurement

In order to describe the machining potential of the grinding wheel active surface, the *Shos* parameter was used. This parameter combines information about the height of surface points and the sharpness of the slope in the vicinity of these points. Its importance results from the fact that highly located and sharp vertices are the most important for the grinding process.

Tool wear usually begins with the abrasion of active grain vertices. As a result, the highest corners become blunt. Therefore, changes in the *Shos* parameter may be used to assess the condition of the grinding wheel active surface and to describe the progress of wear.

The *Shos* parameter should be determined for vertices located above a selected level. The step used to determine the discrete value of this parameter should not be greater than 0.2 of the average size of the base of vertex elevations above the adopted level. The cut-off level should be approximately equal to two average thicknesses of the removed layers or above 0.5 of the grinding depth.

The *Shos* parameter was determined from the measured areal topography of the grinding wheel active surface for surface points located above the adopted threshold level $z_{h}$, which separates potentially active grain vertices from the remaining part of the tool surface. In this study, the threshold level was related to the grinding wheel infeed used in the experimental topography analysis [73], i.e., $z_{h}=max\left(z\right)-a_{e}$, where $a_{e}=20\;\mu m$. The definition of *Shos* follows the metrological approach proposed for the assessment of the machining potential of abrasive tools and extends the height-and-sharpness description of protrusions by including the orientation of abrasive grain ridges relative to the main grinding direction. For each analyzed point $\left(x_{i},y_{j}\right)$, a four-field analysis window *X* was adopted:

(1) $$X=\{\left(x_{i-k},y_{j}\right),\left(x_{i+k},y_{j}\right),\left(x_{i},y_{j-k}\right),\left(x_{i},y_{j+k}\right)\}.$$

For points satisfying $z\left(x_{i},y_{j}\right)>z_{h}$, the height-and-sharpness operator $\omega \left(x_{i},y_{j}\right)$ was calculated as:

(2) $$\omega \left(x_{i},y_{j}\right)=z{\left(x_{i},y_{j}\right)}^{4}-\prod_{p\in X}z\left(p\right)\cdot g\left(x_{i},y_{j}\right),\;\;z\left(x_{i},y_{j}\right)>z_{h},$$

where the penalty function $g\left(x_{i},y_{j}\right)$ describes the local shape of the protrusion:

(3) $$g\left(x_{i},y_{j}\right)=1-\frac{\frac{1}{4}\sum_{p\in X}\left|z\left(x_{i},y_{j}\right)-z\left(p\right)\right|}{max\left(z\left(x_{i},y_{j}\right),z\left(p\right):p\in X\right)}.$$

To include the orientation of the abrasive grain ridge, the operator $U\left(x_{i},y_{j}\right)$ was introduced:

(4) $$U\left(x_{i},y_{j}\right)=\omega \left(x_{i},y_{j}\right)+z{\left(x_{i},y_{j}\right)}^{2}\prod_{p\in K}z\left(p\right),$$

where K⊂X is the set of points located in the plane perpendicular to the main movement direction. The local value of the *Shos* parameter was then calculated as:

(5) $$Shos\left(x_{i},y_{j}\right)=sgn\left(U\left(x_{i},y_{j}\right)\right){\left|U\left(x_{i},y_{j}\right)\right|}^{0.5}.$$

The *Shos* values may be averaged with respect to the potentially active grain vertices or to the entire analyzed active surface of the abrasive tool:

(6) $$Shos_{ag}=\frac{\sum_{q\in N_{a}}Shos\left(q\right)}{card\left(N_{a}\right)\;\Delta x\;\Delta y},$$

(7) $$Shos_{as}=\frac{\sum_{q\in N_{a}}Shos\left(q\right)}{\left(N_{x}-2k\right)\left(N_{y}-2k\right)\;\Delta x\;\Delta y},$$

where $N_{a}=\{q:z\left(q\right)>z_{h}\}$ is the set of surface points located above the threshold level, Δx and Δy are the sampling intervals, and $N_{x}$ and $N_{y}$ are the numbers of measured points in the x and y directions, respectively. High values of $Shos\left(x_{i},y_{j}\right)$ indicate high and sharp protrusions with favorably oriented active cutting ridges. A decrease in *Shos* values indicates blunting, wear of active grain vertices, adhesion of workpiece material, or clogging of inter-grain spaces. Therefore, the *Shos* parameter was used in this study as a local indicator of the machining potential and wear state of the grinding wheel active surface. To assess the usefulness of the *Shos* parameter in differentiating the active surface condition of abrasive tools, three grinding wheels from ANDRE ABRASIVE ARTICLES were investigated. The tested wheels represented three different concepts of active surface structure: a conventional grinding wheel, a grinding wheel with modified spatial structure and increased porosity, and a grinding wheel containing special abrasive grains with a spatially developed structure. The wheel characteristics were as follows: A: 1-250 × 25 × 76.2-99A100K7VTE10-35; B: 1-250 × 25 × 76.2-99A100K7IVTE10-35; and C: 1-250 × 25 × 76.2-99AY100K7VTE10-35. Wheel A was used as a reference tool, wheel B represented the effect of increased structural porosity, whereas wheel C represented the effect of spatially developed abrasive grains, which may act as aggregate-type cutting elements. The operational tests were carried out with a grinding speed of vs = 35 m/s, a longitudinal feed speed of vft = 25 m/min, a transverse feed of ap = 0.5 mm/pass, and a grinding wheel infeed of ae = 100 µm. The dressing infeed ad was 0.1–0.15 mm. The workpiece material was X153CrMoV12 tool steel, also designated as NC11LV.

Replica methods were used to study the topography of the abrasive tools. The RepliSet system by Struers was used, with a nominal resolution of 0.1 µm. The changes in the local values of the *Shos* parameter were analyzed before grinding and after 30 min of grinding. Spectral methods were also used in the analysis of the grinding wheel active surface. Power spectral density determined from surface values makes it possible to observe changes in the spatial components of the surface after grinding. This analysis may support the interpretation of active surface changes and may be used together with the *Shos* parameter in tool wear diagnostics.

### 2.3. Numerical Model of Micro-Cutting

In order to explain the way in which the workpiece material flows during micro-cutting, simulations of cutting with a single abrasive grain and with an abrasive aggregate were carried out. The purpose of this part of the study was to compare two different abrasive forms and to show how the geometry of the aggregate may support the development of new abrasive tools.

The simulations were carried out using the DEFORM 3D V13.1 software environment. In the workpiece material, translational and rotational degrees of freedom were removed for the nodes located at the base of the material. Boundary conditions were introduced for the velocity of the grain and the aggregate. The velocity value was 10 m/s.

Several simplifications were introduced in the numerical model. The mechanical properties, deformation, fracture, and wear of the abrasive grain and the abrasive aggregate were not considered. Both abrasive bodies were assumed to be rigid since their material is much harder and stiffer than the Ti-6Al-4V workpiece material. The main objective was to analyze the deformation and flow of the workpiece material rather than the mechanical response of the abrasive bodies. The workpiece material was modeled as homogeneous in order to separate the influence of abrasive geometry from microstructure-related effects. Coolant action, bond deformation, abrasive wear, heat transfer effects, and full thermo-mechanical coupling were not considered in the present model. Therefore, the presented results should be interpreted as a comparative micro-cutting analysis aimed at evaluating the influence of a single abrasive grain and an abrasive aggregate on material flow, chip formation, and lateral pile-up formation under identical cutting depth and boundary conditions.

### 2.4. Constitutive and Damage Models

The simulation of micro-cutting processes requires the selection of a material model that describes the behavior of the workpiece material under large strains, high strain rates, and temperature changes. For this reason, the Johnson–Cook model (8) was used:

(8) $$\sigma =\left(A+B\varepsilon_{p}^{n}\right)\left[1+Cln\left(\frac{{\dot{\varepsilon }}_{p}}{{\dot{\varepsilon }}_{0}}\right)\right]\left[1-{\left(\frac{T-T_{amb}}{T_{melt}-T_{amb}}\right)}^{m}\right]$$

where A is the initial static yield strength; B is the plastic strength parameter; ε_p_ is the effective plastic strain; n is the strain hardening exponent; C is the material parameter describing the influence of strain rate; ${\dot{\varepsilon }}_{p}$ and ${\dot{\varepsilon }}_{0}$ are the effective and reference strain rates; T, T_amb_, and T_melt_ are the current, ambient, and melting temperatures; and m is the thermal softening exponent.

The material constants were adopted for the Ti-6Al-4V titanium alloy. The following values were used: A = 782.7 MPa, B = 498.4 MPa, C = 0.028, *n* = 0.28, and m = 1.0. These constants describe the influence of plastic strain, strain rate, and temperature on the flow properties of the material.

In order to simulate material separation, the Cockroft–Latham damage criterion was used. In this model, the damage coefficient increases with material deformation, and material separation occurs when the coefficient reaches the critical value. The damage criterion (9) was written as:

(9) $$D=\int \frac{\sigma^{*}}{\sigma }\;d\varepsilon ,\;\;D_{f}=820$$

where σ* is the maximum tensile principal stress, σ is the effective stress, and dε is the effective strain increment. The critical damage value was set to Df = 820. According to the Cockroft–Latham criterion, cracking and separation of the workpiece material occur when the damage integral reaches the critical value.

### 2.5. Mesh and Contact Conditions

The workpiece material was discretized using 140,000 tetrahedral finite elements. In order to optimize calculation time and improve the quality of results in the contact zone, the Autoremesh function was used. This function increases the mesh density in the region between the abrasive grain or aggregate and the workpiece material.

The selection of contact and friction conditions is also important in machining simulations. A constant shear friction model was used. The frictional stress was defined as:

(10) $$f_{s}=mk$$

where f_s_ is the frictional stress, k is the shear yield stress, and m is the friction factor. Previous studies show that the selection of the constant shear friction value should take into account variables such as rake angle and thermal effects. On this basis, the friction condition was assumed as f_s_ = 0.8.

### 2.6. Geometrical Parameters of the Abrasive Grain and Aggregate

**Figure 2:**
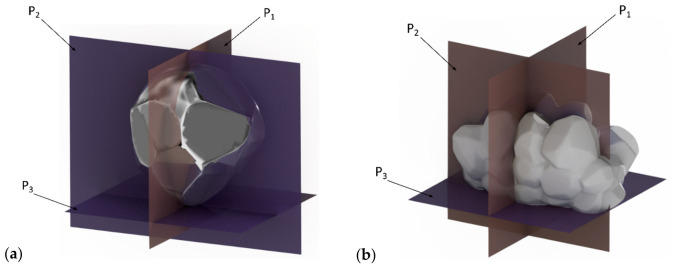
The geometrical features used in the micro-cutting analysis: (**a**) single abrasive grain; (**b**) abrasive aggregate.

The geometrical parameters of the abrasive grain and the abrasive aggregate were determined in three mutually perpendicular planes affecting the material removal process. In plane P1, parallel to the cutting direction, the rake angle γ was determined. In plane P2, perpendicular to the cutting direction, the apex angles ε_1_ and ε_2_ were determined. In plane P3, parallel to the workpiece surface, the width of the cutting zone b_z_ and the length of lateral material displacement b_b_ = b_1_ + b_2_ were determined (Figure 2). The parameters were determined for a constant depth of cut h = 2 µm.

**Figure 3:**
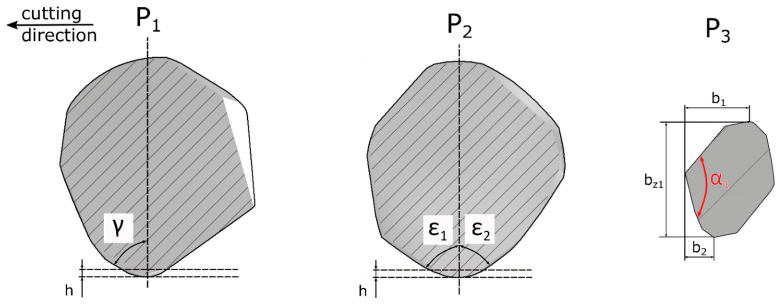
The geometric properties of the abrasive grain determined in the section planes P1, P2, and P3 defined in Figure 2.

The abrasive grain was characterized by a wedge-shaped rake face with α_1_ = 120° (Figure 3). For the penetration depth h = 2 µm, the rake angle was γ = −78°. The grain had a narrow cutting edge b_z_. The sum of the two lateral edges b_1_ and b_2_ was only slightly smaller than b_z_. Such geometry may favor lateral displacement of the workpiece material and the formation of pile-ups.

**Figure 4:**
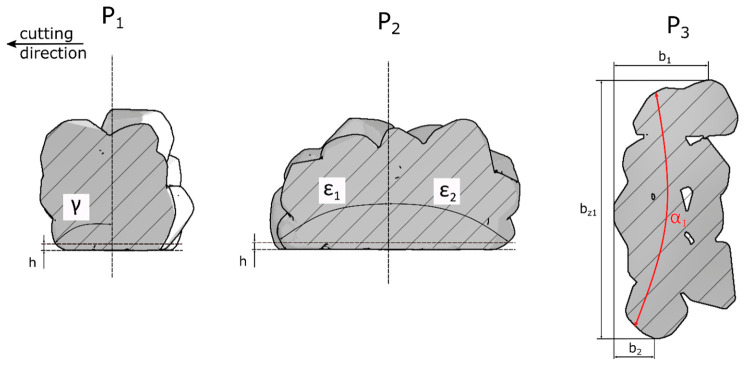
The geometric properties of the abrasive aggregate determined in the section planes P1, P2, and P3 defined in Figure 2.

The abrasive aggregate was characterized by a convex rake face with α_1_ = 160°. For the penetration depth h = 2 µm, depending on the section of the grain in the aggregate, the rake angle ranged from γ = −65° to γ = −73°. The aggregate had a wide cutting edge b_z_, while the sum of the lateral edges b_1_ and b_2_ was much smaller than bz (Figure 4). This feature is desirable because it supports chip formation and reduces the ability of the workpiece material to move sideways.

For the single grain, at a depth of cut of 2 µm, b_1_ = 21 µm, b_2_ = 8 µm, and b_z_ = 30 µm, the ratio b_b_/b_z_ was 0.96. For the aggregate, at the same depth of cut, b_1_ = 49 µm, b_2_ = 22 µm, and b_z_ = 133 µm, the ratio b_b_/b_z_ was 0.53. These values show that the aggregate has a much wider cutting zone in relation to the lateral displacement edges than the single grain.

### 2.7. Material Removal Efficiency

The material removal efficiency of the abrasive grain and the abrasive aggregate was evaluated using the coefficient k_r_ (11). This coefficient takes into account the volume of material removed in the form of a chip and the volume of lateral pile-ups formed on both sides of the cutting edge:

(11) $$k_{r}=\frac{V_{G}}{V_{G}+V_{R}}$$

where V_R_ is the volume of lateral pile-ups and V_G_ is the volume of material removed by the cutting edge.

It is assumed that k_r_ = 1 when the entire volume of material is removed in the form of a chip and the volume of lateral pile-ups is equal to zero. This coefficient was used to compare the effectiveness of material removal by the single abrasive grain and by the abrasive aggregate.

## 3. Results and Discussion

### 3.1. Theoretical Background and Key Process Characteristics

#### 3.1.1. Economic Importance of Improving and Optimizing Grinding Processes

According to a study by MarketResearch.com, the global market for grinding wheels was valued at USD 5.2 billion in 2022. Forecasts indicate that by 2029 it will reach almost USD 7 billion, with an annual growth rate of 3.9%. These forecasts also include assessments of the global market for grinding wheels made of polycrystalline diamond, polycrystalline cubic boron nitride, and monocrystalline diamond, taking into account the programs of key global manufacturers in this field, such as KLINGSPOR Abrasives, Saint-Gobain, DSA Products, Kinik, DK Holdings, Disco, Tokyo Diamond Tools Mfg. Co., Ltd., Asahi Diamond Industrial, and Kure Grinding Wheel.

Assuming that the value of grinding operations performed using abrasive tools over their service life is twenty times greater than the value of the tools themselves, the global value of machining operations reaches approximately USD 100 billion annually. On this basis, the effects of improving and optimizing these operations may be estimated. Assuming that, as a result of optimization, product value increases by 0.5% and production costs decrease by 1%, then, under comparable conditions and time periods, the annual global effect would correspond to a value of USD 1.5 billion.

It should also be taken into account that the share of precision machining is increasing at a rate slightly higher than the increase in sales of abrasive tools, at a level slightly exceeding 4% per year.

This leads to the conclusion that investment in the research and development of abrasive tools, as well as in the improvement of machining processes and the use of new structural materials, is a desirable strategy for the development of many companies. Effective support for manufacturers of abrasive tools and access to new knowledge, in the form of the latest reports and publications, may increase the technological and economic effects of improving and optimizing grinding processes.

**Figure 5:**
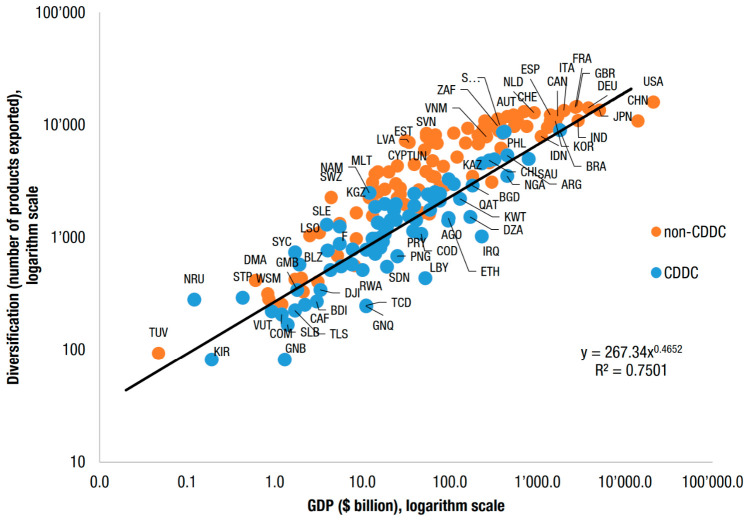
The relationship between the number of exported products and gross domestic product, shown on logarithmic scales [77].

Innovations concerning new abrasive tools belong to product innovations, which support economic diversification through the development of sectors with higher productivity. Economic diversification is correlated with gross domestic product [1]. Figure 5 presents this relationship with respect to the effects of all product innovations [2]. It may be assumed that abrasive tools, as a technological component in precision manufacturing processes, show a similar pattern of influence.

#### 3.1.2. Analysis in the Field of Modeling and Optimization of Grinding Processes

**Figure 6:**
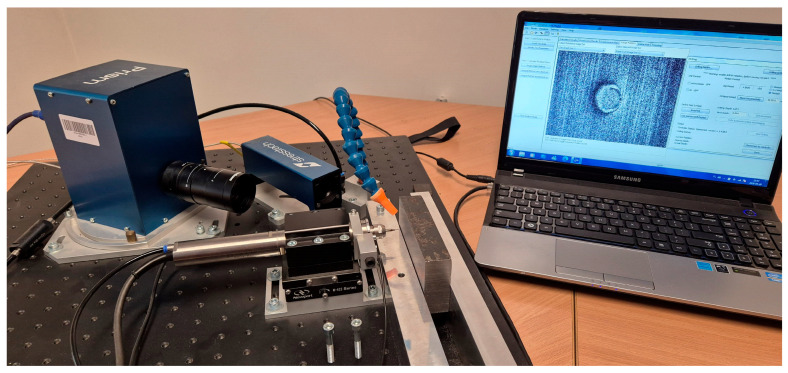
The PrismRS system used for residual stress measurement based on the interferometric hole-drilling method.

Residual stresses were measured by means of the interferometric hole drilling method, which was used in PrismRS software. This method involves the combination of material removal and optical detection of deformation of the surface of the material (Figure 6). It is based on capturing the strains of the surface resulting from the release of stress during material removal. The original condition of the material corresponds to certain residual stress distribution in the body. The drilling of a small hole disturbs the equilibrium of local stresses, leading to stress release and creating the displacement field in the neighborhood of the drilled hole. Then, this deformation field is captured using an interferogram.

In [78], the results of introducing compressive stresses into the surface layer by grinding with a low peripheral speed of the grinding wheel under conditions of small machining depths were presented.

The importance of temperature field models in the selection of grinding parameters was emphasized in [79]. The problems of validating thermal models of the contact zone were described, and the results obtained using an infrared measurement system were presented. Analyses of thermal transformations in the grinding zone were carried out in [80]. The authors developed a heat flux model taking into account process parameters and the characteristics of the grinding wheel.

Modeling of the grinding process and the development of foundations for predicting process characteristics have long been an effective method for creating optimization models [81]. In previous studies, many different models have been developed, especially models of grinding forces [82]. The first model taking into account many process features [83] was a relationship describing the connection between force and process parameters. It was further developed in many other works, including [84,85].

The beginnings of modeling consisted in improving empirical relationships, including through the use of artificial neural networks [86]. In subsequent works, the possibilities of creating models using multidimensional analyses were used [87]. In other studies, energy aspects of micro-chip formation and friction forces in the cutting zone were separated [88,89].

The accuracy of grinding force modeling was increased as a result of works in which the topographic features of the grinding wheel were taken into account [90]. Stochastic modeling contributed to the development of modeling methods [91]. This made it possible to include distributions of grain locations and to determine the sum of forces from individual grains. Taking grain trajectories into account [92] extended the application range of the developed models.

**Figure 7:**
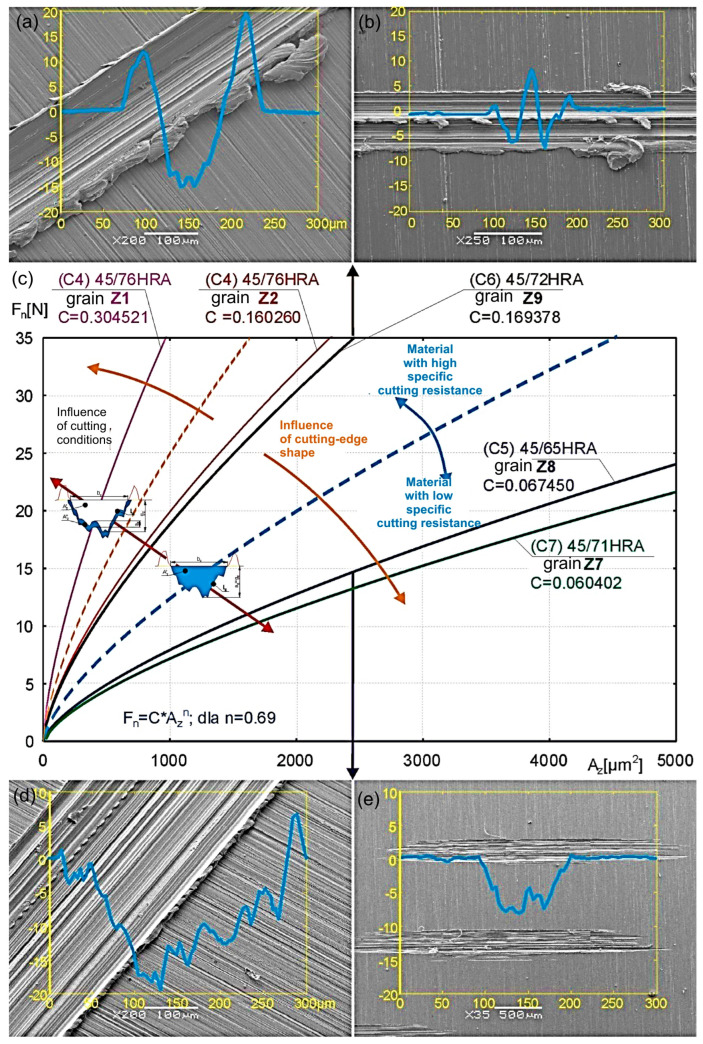
A comparison of the normal component of the micro-cutting force for a single grain, Fn = C·Az^n^, for different shapes of the cross-section of the removed layer: (**a**,**b**,**d**,**e**) SEM images of scratch tracks with overlaid transverse profiles; (**c**) relationship between the normal force Fn and the cross-sectional area Az of the removed material layer for selected abrasive grains [95].

The analysis of the influence of the cross-sections of removed layers on grinding forces and energy [93,94] still does not explain the relationship between the volume of pile-ups and the width of the layers removed by individual grains. This is because material removal efficiency depends not only on the cross-sections of the removed layers, but also on the relationship between the width and thickness of these layers, as well as on the profile of the layer (Figure 7). Figure 7 shows the morphologies that were obtained from scanning electron microscopy (SEM) using a Phenom World SEM. This device is capable of magnifying surface morphologies, scratch morphologies, and displacement traces obtained in micro-cutting. F_n_ is the force experienced when micro-cutting, and A_z_ refers to the cross-sectional area of the layer of material being removed. The empirical equation F_n_ = C·A_z_^n^ expresses the effect of force as a function of the cross-sectional area of the material removed, where C is a constant that depends on the morphological characteristics of the grains, material of workpiece, and machining conditions, and *n* = 0.69 is used for analyzing the results shown in the current experiments. In the figure, Z1, Z2, Z7, Z8, and Z9 denote the grains used for carrying out the scratch test experiments, whereas the numbers after HRA in parenthesis refer to the hardness of the workpiece material. Blue lines overlaid on the SEM images demonstrate transverse profiles of the formed grooves.

Graphically presented in Figure 7 is the correlation between the normal component of the micro-cutting force F_n_ and the cross-sectional area A_z_ of the removed material layer for a group of abrasive grains subjected to different cutting conditions. The graph can be characterized mathematically using the empirical equation F_n_ = C·A_z_^n^, where C is a coefficient that depends on the grain shape, material type, and cutting conditions, whereas an exponent *n* = 0.69 is considered valid for the analysis performed in this research. The grains used during scratch testing were marked as Z1, Z2, Z7, Z8, and Z9, whereas the hardness of the machined material was within the range of 65–76 HRA. In addition, one can see that the different gradients of the graphs imply that besides the area of the cut off layer, the micro-cutting force is determined by the cutting edge geometry and resistance of the material to cutting. Thus, steeper gradients reflect a high value of the specific cutting resistance and adverse cutting conditions; conversely, flat gradients stand for effective material machining. Micrographs around the graph depict several scratches created by single abrasive particles. These transverse profiles reveal that the grain morphology determines the shape of the groove and lateral displacement of the material.

**Figure 8:**
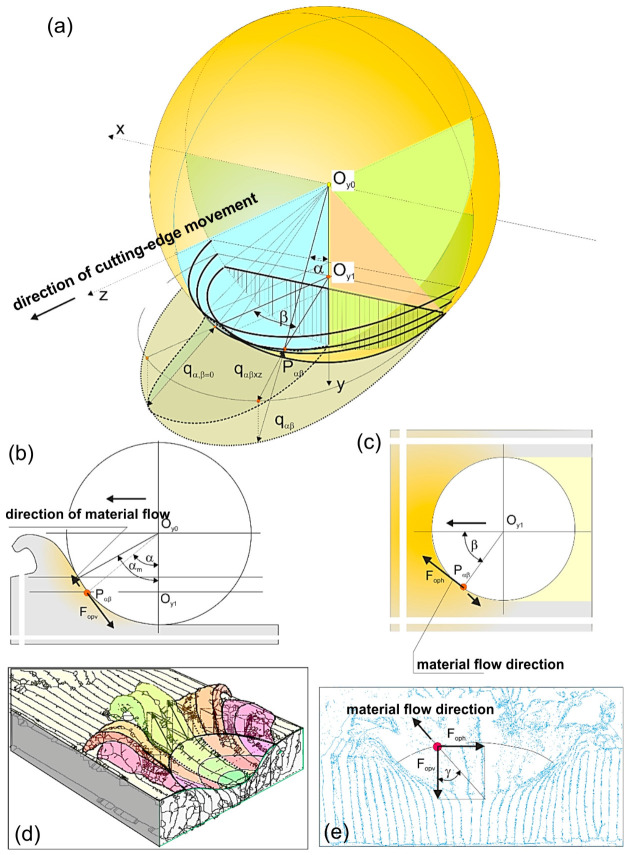
A scheme for the analysis of material displacement resistance during micro-cutting with an abrasive grain vertex: (**a**) a spatial model of the active contact point Pαβ and angular parameters α and β; (**b**) a side-view scheme of material flow and the vertical displacement resistance force Fopv; (**c**) a top-view scheme of material flow and the horizontal displacement resistance force Foph; (**d**) simulated material flow and pile-up formation around the groove; (**e**) the distribution of material flow directions and displacement resistance force components.

In [96], advanced models of energy and heat fluxes generated by individual grains distributed according to a specified distribution on the grinding wheel surface were presented. The validation of temperature fields and grinding forces by comparing FEM results with experiments demonstrated the usefulness of the developed models for predicting process characteristics. In the analysis of lateral material flows in micro-cutting, physical models and analyses of the resistance to lateral displacement of material relative to the grain path are also useful (Figure 8).

FEM modeling and the results of experimental studies [97] made it possible to determine the influence of the geometrical features of the active grain vertex on pile-up formation and the beneficial effects of limiting lateral material flows in micro-cutting with grains having a wide rake face and small angles of its inclination from the plane perpendicular to the grain path. Figure 8 illustrates symbols that represent the geometrical location of the active point of contact, the flow directions of materials at that point of contact, and also the vertical and horizontal forces resisting material flow.

Figure 8 depicts the geometrical model adopted to assess the resistance against lateral material displacement provided when performing micro-cutting with a vertex of the abrasive grain. The geometrical model describes the interaction between the moving cutting edge and workpiece material with regard to the local system of coordinates (x, y, z), whereas the arrow shows the direction of the cutting edge movement, and the opposite direction is shown in the section views as an illustration of material flow. Point P_αβ_ is the interface between the abrasive grain and the displaced material that is being analyzed. The point is characterized by the values of the angles α and β, which define the location of the active point of the grain surface longitudinally and horizontally, respectively. Centers of references are represented by the letters O_y0_ and O_y1_, and the corresponding components of the material displacement or the sections are denoted by q_αβ_, q_α,β=0_, and q_αβxz_. Components of forces acting on material displacement vertically and horizontally are F_opv_ and F_oph_, respectively. Figure 8 also presents the resulting flow of material and pile-up around the groove. Thus, the shape and orientation of the abrasive grain vertex determine the direction of material displacement, its intensity, and the required force for forming a groove. For many years, research has been conducted on the application of abrasive tools with discontinuous active surfaces. In [98], a methodology for designing such tools was developed and an analysis of temperature fields in the grinding zone was carried out. The developed models and conducted analyses confirmed a beneficial decrease in temperature in the grinding zone.

Methods supporting abrasive machining with the use of additional kinematic and dynamic interactions are also applied. The use of ultrasonic assistance in the dry grinding process of 42CrMo4 steel showed a beneficial effect of this machining method on reducing surface roughness and reducing grinding force by 60% [99].

#### 3.1.3. Grinding Process Characteristics Important for Creating New Solutions and Improving Abrasive Tools

Important process features, on which the specific energy of machining as well as the physical properties and geometrical structure of the formed surface depend, include the formation of pile-ups around traces produced by active abrasive grains, the number of grains shaping a given surface fragment, and the shape and dimensions of the grinding zone. These features depend on the properties of the grinding wheel, the properties of the workpiece material, and the grinding parameters. In this section, the influence of grinding wheel characteristics on grinding process results is first assessed.

**Figure 9:**
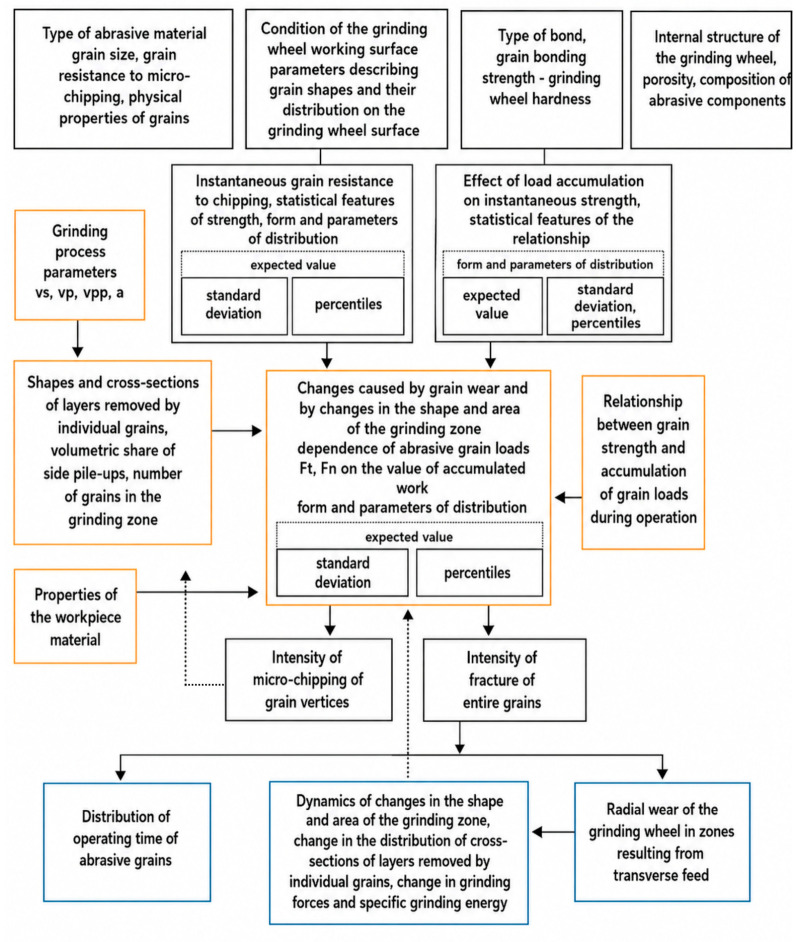
A scheme for the simulation of abrasive grain wear and changes in the shape and position of the grinding zone.

The probability of contact between grains on the surface of the abrasive tool and the workpiece material with a given surface topography depends on the statistical features of the distributions of grain vertices and ordinates of the machined surface, the thickness of the removed layer, the kinematic features of the process, and the compliance of the tool. The results of simulation studies (Figure 9) of the abrasive grain wear process and changes in the shape and position of the grinding zone during surface grinding with the grinding wheel periphery are presented below.

The following data were assumed: grinding speed 35 m/s; longitudinal feed speed vp = 0.3 m/s; transverse feed vpp = 4 mm; grinding wheel diameter D = 250 mm; grinding wheel height H = 10, 20, 30, 40, and 50 mm; and average abrasive grain size az = 0.2 mm, corresponding to grain number 80. The average distance between grains was dz = 1.2·az = 0.24 mm. For these data, with H = 40 mm, the circumferential surface area of the grinding wheel was Aw = 31,416 mm^2^, and the number of grains on the active surface of the grinding wheel was Lz = 545,415. The St parameter of the workpiece surface in the grinding zone was 30 μm, and the standard deviation of the surface ordinates was sdp = 3.41 μm.

The St parameter of the grinding wheel surface was 92 μm, and the standard deviation of the position of the grain vertices was sdw = 18 μm. The highest vertex of the workpiece surface was set at a level of 20 μm from the lowest level of the grain vertices, which means that the nominal grinding depth was 0.2 mm. It was verified that the greatest thicknesses of the removed layer along the path of grain contact with the workpiece material averaged 7 μm. The relationship between the thickness of the removed layers and the grinding depth depends on the ratio of the longitudinal feed speed to the main motion speed and on the distance between active grains.

**Figure 10:**
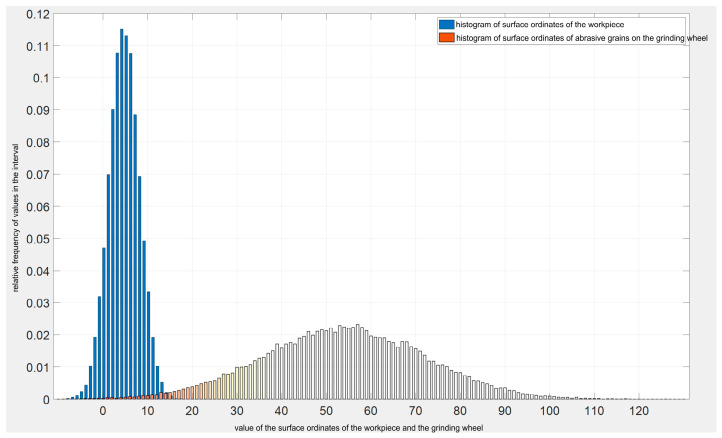
Histograms of the ordinates of the workpiece surface and of the grain surface on the grinding wheel.

The number of grains on the circumferential surface of the grinding wheel with a width of vpp = 4 mm was 54,541, the number of active grains in this zone during one revolution of the grinding wheel was 213, and the share of active grains was 0.4%. A comparison of the position of points on the workpiece surface and points on the surface of abrasive grains on the grinding wheel is presented in Figure 10.

**Figure 11:**
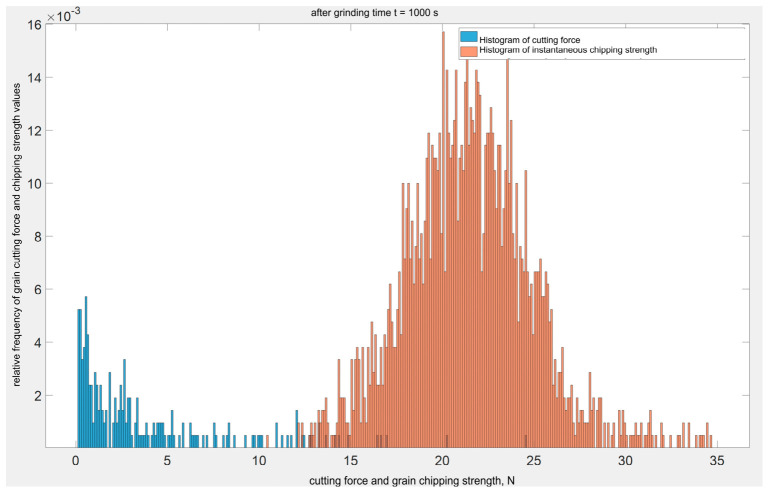
Histograms of cutting force and instantaneous fracture resistance of grains.

The conducted analyses also concerned the relationship between the cutting force acting on individual grains and the resistance of grains to fracture. It was taken into account that the forces acting on individual grains depend on the cross-section and shape of the removed layer, as well as on the cumulative loads of the grain up to the considered moment of the process, which influence the wear of the vertex. It was assumed that the accumulation of the effects of work performed by individual grains affects the reduction in their instantaneous fracture resistance (Figure 11).

For very small grinding depths, the probability of contact between grain vertices and the vertices of the machined surface is so low that the share of active grains in the number of grains on the tool surface, for grinding depths for example smaller than 2 μm, is below 0.2%.

The simulation and experimental studies concerning micro-cutting and grinding with small depths lead to several important conclusions regarding the design and operation of abrasive tools and the optimization of grinding processes.

Grinding wheels for grinding with small machining allowances, below 10 μm, should have a reduced distance between the vertices of active grains. The use of small machining allowances and the corresponding small grinding depth allows the process energy to be significantly reduced, which favors obtaining more beneficial properties of the surface layer, especially when grinding materials with good thermal conductivity. Limiting the grinding depth makes it possible to reduce the density of heat fluxes, shorten the time of local heat-source interaction, and reduce temperature gradients. The use of small grinding depths reduces forces, which may be important for machining small-sized workpieces with high compliance.

**Figure 12:**
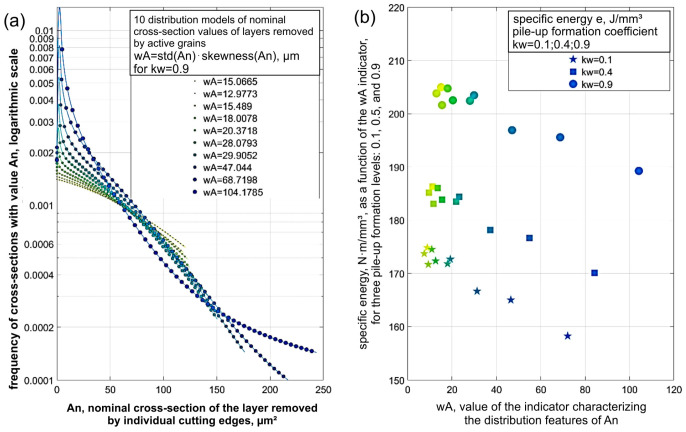
The results of modeling the influence of the distribution of cross-sections of layers removed by individual grains on the specific grinding energy: (**a**) model distributions of nominal cross-sections An of layers removed by individual cutting edges; (**b**) specific grinding energy as a function of the indicator wA characterizing the non-uniformity and asymmetry of the An distribution, determined for different pile-up formation coefficients kw.

However, the use of small grinding depths reduces the share of active grains that shape the machined surface. This may be prevented by reducing the dispersion of the operating radii of grain vertices through the use of additions in the form of abrasive micro-aggregates, polycrystalline grains forming many sharp corners with aligned positions, or bonds with sub-locally increased compliance. Reducing the dispersion of the operating radii of active grains is beneficial for forming surfaces with low roughness; however, it results in a certain increase in specific energy (Figure 12).

**Figure 13:**
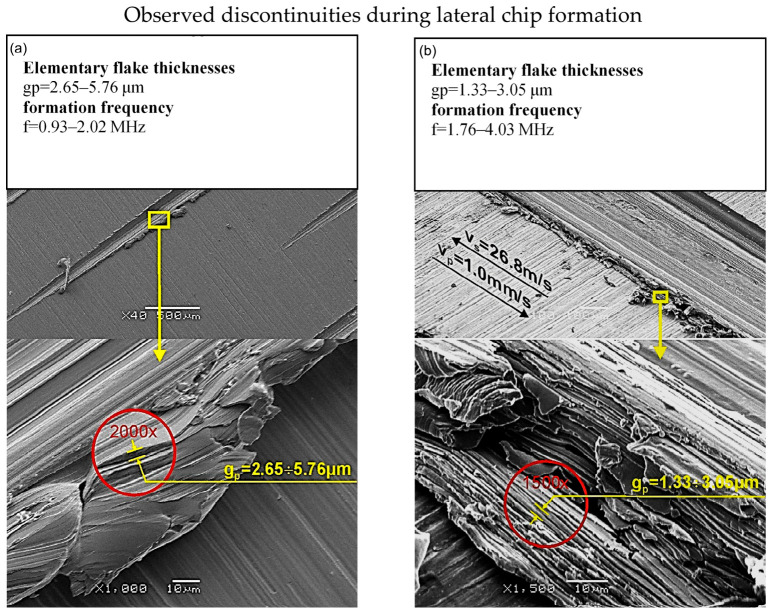
Illustrations for the analysis of lateral pile-ups in micro-cutting processes with abrasive grains during grinding: (**a**) lateral pile-up morphology with observed discontinuities and elementary flake thickness gp = 2.65–5.76 μm, corresponding to the formation frequency f = 0.93–2.02 MHz; (**b**) lateral pile-up morphology with smaller elementary flake thickness gp = 1.33–3.05 μm and higher formation frequency f = 1.76–4.03 MHz.

The specific energy depends on the form of the distribution of removed-layer cross-sections at a fixed material removal rate. In the analysis of the results shown in Figure 12, the product of the standard deviation and skewness of the An distribution was adopted as the indicator wA, which characterizes the non-uniformity and asymmetry of the layers removed by active grains. The left plot shows ten model distributions of nominal cross-sections An, while the right plot presents the calculated specific energy as a function of wA for different pile-up formation coefficients kw. The results indicate that, for a given value of kw, changes in the distribution of An affect the specific grinding energy, whereas increasing the contribution of pile-up formation increases the energy demand of material removal. Despite this energy-related aspect, reducing the variation in the cross-sections of layers removed by individual grains remains a desirable strategy, because it improves the topography of the ground surface and reduces its local variability. The use of small grinding depths unfavorably increases the share of lateral pile-ups in material displacement (Figure 13), which may be limited by increasing the resistance to lateral material flows. Lateral pile-ups, similarly to micro-chips, are characterized by a discontinuous structure in the form of raised plates. From their thickness, cutting speed, and degree of upsetting, the frequency of their formation may be calculated.

**Figure 14:**
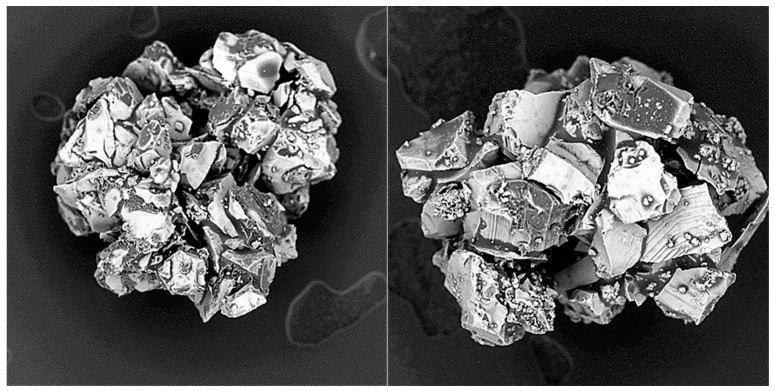
Images of abrasive micro-aggregates containing grains of special noble electrocorundum [100].

An increase in the width of the removed layer often occurs spontaneously as a result of fractures of the abrasive grain vertex (Figure 14). Despite an increase in unit force, this is a beneficial event, because increasing the width of the removed layer reduces the ratio of pile-up volume to the volume of removed material.

This effect can be achieved permanently by using abrasive aggregates with shapes and compositions depending on the intended application of the tools. The use of abrasive micro-aggregates replaces the preparation of micro-discontinuities on the active surface, because it makes it possible to increase the porosity of the grinding wheel. This becomes possible due to the larger and more developed surface area of the aggregates, which means that bonds enabling the use of higher porosity may be applied.

As shown in Figure 13, lateral pile-ups created as a result of micro-grinding are not continuous but rather characterized by discrete structure. The displaced material of the workpiece creates an aggregate of thin platelets formed near the grooves at the expense of discontinuous material movement. This morphology suggests that besides being simply displaced laterally, the material gets periodically raised, compressed, and locally split during the abrasive grain movement on the surface. It can be seen that elementary flakes with different thickness and their respective occurrence rates also confirm the cyclic character of lateral pile-up formation. Thinner flakes occur more often than the thicker ones. It can therefore be concluded that the process of lateral material displacement while grinding is extremely dynamic depending on the local geometry of the abrasive grain apex, its velocity, and resistance to material displacement. Thus, considering this material flow pattern shown in Figure 13, it is possible to suggest that the control of lateral material flow is one of the means of increasing material removal efficiency and ensuring stable grinding surface formation. In grinding processes with small depths, other methods of increasing process efficiency may also be used, concerning the optimization of parameters or dedicated kinematic systems. High values of longitudinal feed speed may be used, which reduces the share of entry and exit zones and increases the length of the cutting trace. In such a case, the transverse feed should be very small in order to obtain a sufficiently large number of micro-cutting traces per unit area.

For plane micro-grinding, dedicated kinematic systems may be used to ensure a low transverse feed speed, crossing of machining traces, a long grinding zone, and slow penetration of grains along a long grinding path.

### 3.2. Development Directions for Advanced Abrasive Tools

The analyses presented in the previous section show that the most important objectives of improving abrasive tools are as follows:

- Reducing the specific grinding energy and temperature in the grinding zone;
- Reducing surface roughness and decreasing local differences in surface topography, and, in more advanced applications, obtaining a dedicated geometrical structure of the surface;
- Increasing tool life, taking into account the wear of grain vertices and changes in their shape;
- Increasing machining efficiency and reducing machining costs.

These objectives can be achieved by reducing the volume of pile-ups through the use of tool structures containing different types of abrasive aggregates, with spherical, ribbon-like, or toroidal shapes. This causes the width of the removed layers to increase at the same thickness of the removed layer.

Another method is to increase the uniformity of the cross-sections of layers removed by grain vertices in aggregates. It is also beneficial to increase the porosity of grinding wheels, to add fillers, or to obtain higher porosity by relatively reducing the bond bridges connecting abrasive aggregates. This is possible because the aggregate surface is developed and extended.

The bond used in aggregates should have properties which ensure that, during aggregate wear, grains located on flat or slightly concave external fragments of aggregates remain active. The improvement of abrasive tools may also involve cooling systems that ensure better use of the machining fluid. Another direction is the production of regular miniature textures on grinding wheel surfaces, in the form of grooves or bands.

Many manufacturers of abrasive tools are developing bonds that increase the grain holding force [38]. Smaller bond bridges reduce friction energy and the specific grinding energy. Smaller volumes of bond bridges also facilitate the supply of machining fluid. Larger spaces between grains make it easier to store micro-chips while they are still in the grinding zone, which is important when the grinding zone is long. The reduction in grinding wheel mass is also beneficial for process stability.

The improvement of abrasive tools for machining light metal alloys is becoming increasingly important. These materials are difficult to machine because the spaces between grains are easily filled with machining products. Due to their considerable plasticity, larger pile-ups are formed, the energy of plastic deformation is higher, and local variation in the geometrical structure of the surface is observed. This indicates fluctuations in the surface formation process.

These phenomena result from the fact that, in front of the abrasive grain and below the shear surface of the workpiece material, a zone of unstable material displacement is formed. Thin flakes of the workpiece material are produced and permanently pressed into the spaces between grain vertices. This irregularly reduces the time until renewal of the tool surface becomes necessary.

Limiting these unfavorable process features was the aim of studies concerning the development of new abrasive tools with innovative, adaptive structures and special micro-aggregates. In the developed tools, next to abrasive grains of a specified size connected by a bond forming a porous structure, special micro-aggregates are present. These micro-aggregates are composed of grains with dimensions much smaller than those of the basic fraction (Figure 14). Micro-aggregates may also contain abrasive grains made of materials different from those used in the basic grain fraction.

A micro-aggregate is formed by micro-grains connected with a bond whose properties depend on the intended application. The bond of a micro-aggregate may be metallic, resin, ceramic, polyurethane, or polyamide. Depending on the size of the aggregate and the size of the micro-grains, the produced aggregate contains several to several dozen abrasive elements connected by a bond that provides a specified holding force and porosity.

In this tool structure, aggregates and grains of the basic fraction are connected by the main bond. The volume share of the main bond may be lower because the surfaces of the aggregates are large and developed. The use of a specified share of micro-aggregates, for example 30%, contributes to obtaining a machined surface with more favorable stereometric parameter values compared with a conventional grinding wheel. It is also possible to use, in one tool, aggregates containing grains of different types and made of different abrasive materials.

Micro-aggregates ensure stable operation of many grain vertices along the paths of individual aggregates. The cutting edges of grains in the micro-aggregate, arranged at similar operating radii, ensure the repeatability of micro-cutting. In contrast, in conventional grinding wheels, large grain corners form chips of different shapes and structures, which results from the high variability of material removal conditions.

Abrasive aggregates may also contain an addition of superhard grains in tools intended for machining extremely difficult-to-machine materials.

**Figure 15:**
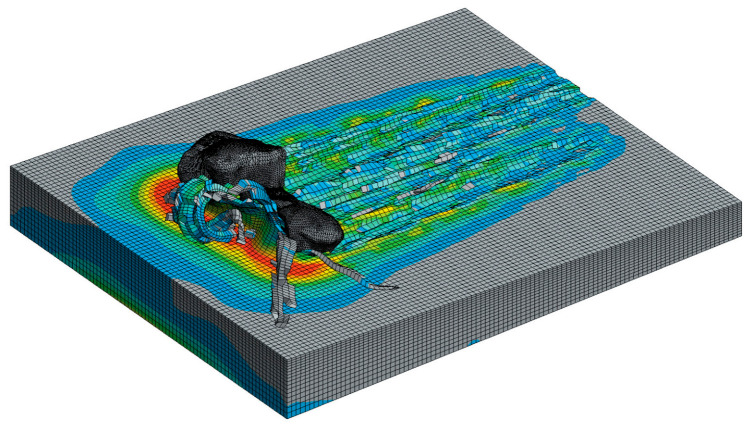
An image of the micro-cutting zone formed by a small abrasive aggregate with a wide micro-cutting zone.

A beneficial effect on reducing the energy consumption of micro-cutting with abrasive aggregates results from their geometrical features and from the forms created as a result of micro-fractures. The long outline of the active part of the aggregate, on which several or more grains are located, significantly hinders and limits lateral material displacement. Small pile-ups are formed and almost the entire removed layer is transformed into a chip in front of the aggregate (Figure 15).

**Figure 16:**
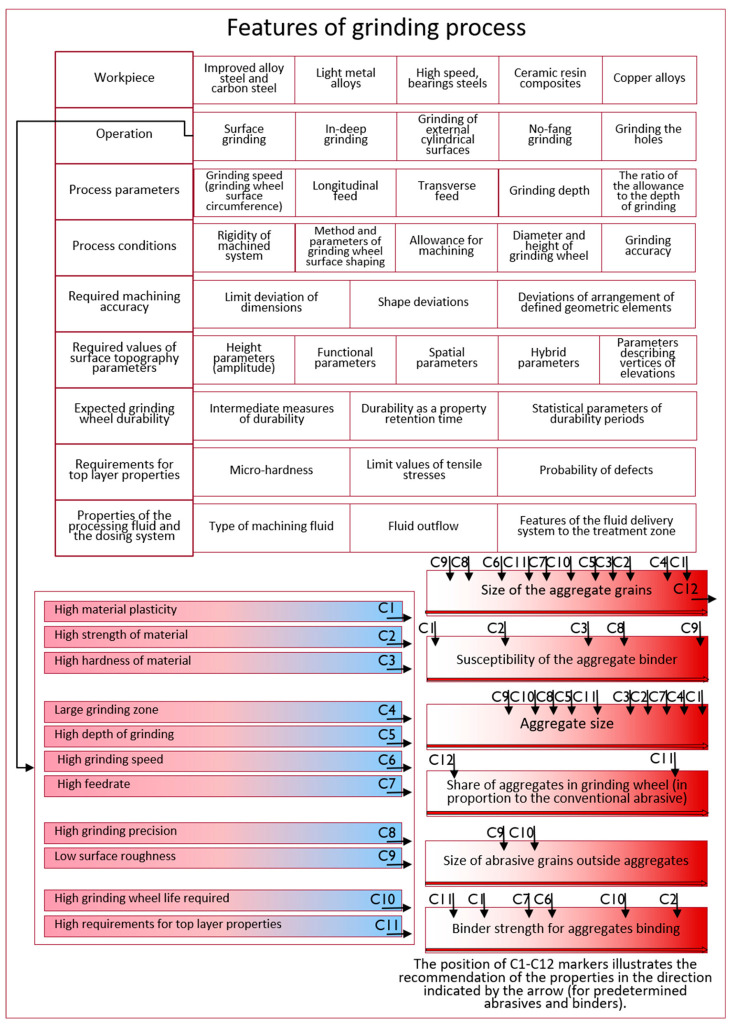
A scheme for selecting the features of a grinding wheel with the addition of aggregates for surface grinding. Symbols C1–C11 denote the features of the grinding operation, and their position in the fields on the right side indicates the selected level of a given grinding wheel property [100].

By introducing dedicated sets of micro-aggregates into the structure of abrasive tools, a new level of selecting tool properties for various machining operations is achieved. For comparison, the properties of conventional abrasive tools can be modified only with respect to the type and size of abrasive grains, the bond, and the structure. In tools with aggregates, it is also possible to differentiate tool properties by selecting the size of micro-aggregates, the size of grains and the properties of bonds forming these aggregates, as well as by differentiating the types of aggregates and their share in the grinding wheel [42,43]. Increasing the possibilities of differentiating tool features naturally makes the selection process for such tools more complex (Figure 16).

The scheme presented in Figure 16 shows that the use of abrasive aggregates significantly extends the range of grinding wheel design variables. In conventional grinding wheels, tool properties are mainly selected by choosing the abrasive material, grain size, bond type, hardness, and structure. In aggregate-based tools, additional design levels are introduced, including the size of aggregate grains, aggregate size, share of aggregates in the grinding wheel volume, size of abrasive grains outside the aggregates, and binder strength inside the aggregate. This makes it possible to adapt the grinding wheel more precisely to the workpiece material, grinding operation, process parameters, required accuracy, surface integrity requirements, and expected tool life.

The main advantage of abrasive aggregates is that several micro-grains may operate together within one active element, forming a wider and more stable cutting front than a single abrasive grain. Such a geometry increases the width of the removed layer at a comparable penetration depth and limits lateral material displacement. As a result, the volume of lateral pile-ups is reduced and a larger part of the displaced material is removed in the form of chips. This mechanism is particularly beneficial in precision grinding, where the small depths of cuts increase the tendency to plowing and side flow of the workpiece material. The use of aggregates therefore improves material removal efficiency, reduces the instability of micro-cutting, and supports more repeatable surface formation. Abrasive aggregates also improve the functional structure of the grinding wheel. Their developed external surface allows the use of lower volumes of the main bond and supports the formation of a more open structure. Larger inter-grain spaces facilitate coolant delivery and chip storage in the grinding zone, which reduces the risk of loading and clogging the active surface. This is especially important during the grinding of materials with high plasticity and thermal sensitivity, where the adhesion of machining products and unstable lateral flow may rapidly reduce the tool’s cutting ability.

Another important benefit is the possibility of controlling the self-renewal mechanism of the active surface. During wear, the external part of an aggregate may expose new micro-grains located at similar operating radii. This supports the stable operation of many cutting vertices along the aggregate path and reduces the dispersion of removed-layer cross-sections. In comparison with conventional grinding wheels, where individual large grain corners may produce highly variable chip shapes, aggregates provide a more controlled micro-cutting mechanism. This contributes to lower surface roughness, reduced local variation in the machined surface topography, and improved grinding stability. The advantages of aggregates are particularly relevant for difficult-to-machine materials, composite materials, light metal alloys, and components requiring high surface integrity. Aggregates may also be designed as hybrid structures containing grains of different sizes or different abrasive materials, including superhard grains. Therefore, abrasive aggregates should not be treated only as an additive increasing porosity, but as functional structural elements that allow the material removal mechanism, tool wear behavior, and surface formation process to be controlled. The use of grinding wheels with abrasive aggregates for grinding the Ti-6Al-4V titanium alloy showed a reduction in grinding forces and specific energy. Wide fronts formed by grains located close to one another are of decisive importance for the efficiency of the material removal process. The most favorable situation occurs when the front line is concave. This may occur when, as a result of wear, the inside of the aggregate becomes a surface with slight concavity.

Grinding the Ti-6Al-4V titanium alloy with grinding wheels containing abrasive aggregates also provides lower roughness of the machined surface. The described abrasive tools are also useful for grinding composite materials and components made of materials with high plasticity and sensitivity to high temperatures. Hybrid abrasive tools with the addition of aggregates and increased porosity largely eliminate the clogging of grinding wheel surfaces. An important property of such tools is the extension of the range of features that can be more precisely adapted to technological tasks.

New directions in the development of advanced abrasive tools include the development of segmented tool structures with abrasive segments having gradient-variable bond properties, high porosity, and sensors embedded in tool bodies.

Another direction is the development of grinding wheel technologies with micro-segments placed on the surfaces of tool bodies. The use of micro-aggregates of different forms, especially spherical or ribbon-like forms with controlled orientation, makes it possible to obtain adaptive grinding wheel structures. The aim is to influence the self-sharpening mechanism of micro-aggregates, which causes the spontaneous formation of favorably positioned fronts composed of micro-grains of aggregates.

Further development directions include producing grinding wheels with local or zonal differentiation of the share and type of abrasive aggregates in the tool volume. Additives may also be introduced together with micro-aggregates to reduce the specific grinding energy, to provide a protective effect on the machined surface, or to facilitate the formation of special structures on the machined surface.

Another important direction is the addition of indicators or sensors to some aggregates for diagnostic purposes and for assessing the condition of the tool. Specialist abrasive tools may also be produced with external layers formed by additive methods in hybrid processes, with single abrasive grains dosed into selected spaces of the porous structure.

### 3.3. Assessment of Grinding Wheel Wear Based on the Shos Parameter

In the literature, many methods of modeling the active surface of abrasive tools have been described, especially for the analysis of their interaction with machined components [44]. These models are useful in simulation procedures and in testing parameters that can be used to differentiate assessments of the tool surface condition.

Among the different modeling methods, procedures based on the description of topography using data from the real grinding wheel surface are visible. Another method consists of developing procedures for the probabilistic description of grains and their distribution on the tool surface. For this purpose, the properties of Voronoi cells may be used, with a generated set of cell areas and vertex positions.

When creating abrasive grain surfaces, it is worth taking into account that the surfaces of abrasive grains contain fragments with different flatness of walls. Often the fractal dimension of vertices is greater than the dimension of depressions formed by the bond. Voronoi cells should also not have excessively differentiated areas, which results from the position and size of the grains.

In creating an effective methodology for evaluating the geometrical structure of the active surface of grinding wheels, and especially the surfaces of grain vertices and the shape of the working surface, the first task is to determine measures useful for assessing the machining potential and identifying forms of wear. In numerous scientific studies and in practical applications of their results, a wide range of standardized parameters is used to evaluate the surface geometric structure. Among the commonly applied parameters, those that integrate data from the entire surface or from large portions of it tend to dominate. However, such parameters often insufficiently reflect the mechanisms involved in surface formation and wear processes. One of the limitations of current diagnostic and predictive approaches in grinding processes is the use of parameters with low information content and limited ability to differentiate information concerning the surface geometric structure. It should be noted that many parameters used to assess the surface geometric structure of grinding wheels gain significance when the information they contain is integrated with information provided by other parameters. However, the method of data integration is important for improving the assessment of changes occurring during abrasive tool wear. The integration of measures in the form of the product of the gradient and the height of abrasive grain summits significantly increases the evaluation contrast and the ability to differentiate the surface conditions of a grinding wheel. In this respect, the *Shos* parameter offers an advantage over other parameters with lower discriminatory capability. For assessing the machining potential of grinding wheels, the ease of interpreting parameter values and relating them to the features and effects of the manufacturing process is also of great importance. The *Shos* parameter addresses the question of whether the summits with the greatest effective radius are subject to wear flattening. Consequently, it enables the assessment of the cause of increasing grinding forces, indicates whether summit wear is dominant, and shows whether this is additionally accompanied by an enlargement of the grinding zone area.

**Figure 17:**
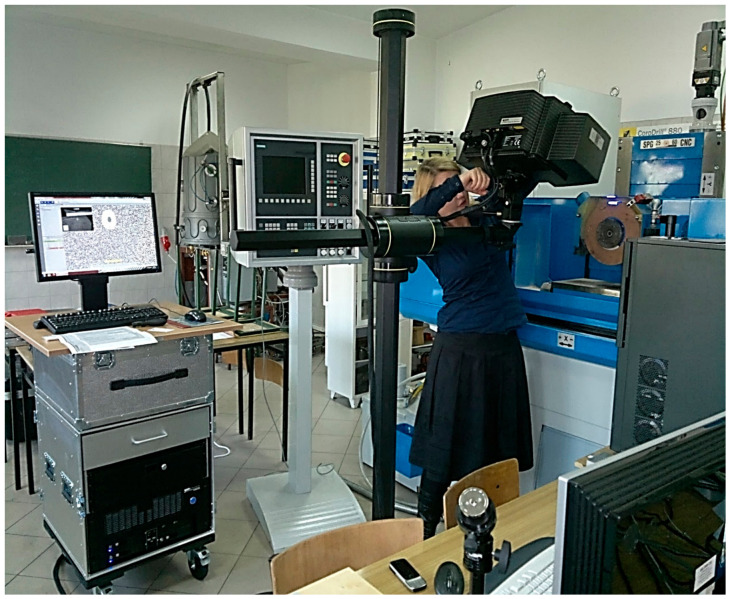
System GOM Atos III for scanning the grinding wheel surface without removing it from the grinder spindle.

The next task is to build a system for determining digital images of grinding wheel surfaces, preferably without removing the wheel from the grinder spindle, using scanning methods (Figure 17), light scattering analysis methods, or replica methods. These tasks precede the determination of tool life criteria.

In the evaluation of stereometric features of grinding wheel surfaces, it is important to extract data that are highly useful technologically and sensitive to changes caused by wear. In the evaluation of surfaces after grinding, parameters are used that are typical for surfaces with motifs whose base size is thousands of times greater than the height of the motif. In the analysis of grinding wheel surfaces, the height of the motif is comparable with the dimension of its base. Ground surfaces have a geometry formed in a permanent way. The grinding wheel surface changes continuously during the process.

In studies of the topography of the active surface of abrasive tools, the important features are therefore the features of grain vertices located above a certain plane, for example at a level from 0.2 to 0.3 of the St parameter value from the highest vertex of the surface.

The grinding wheel contacts the workpiece material with the vertices of grains having the largest operating radii, and the share of active grains is often lower than 1%. The use of Voronoi cells in this analysis makes it possible to determine the distances between the vertices of elevations and the inclination angles of lines connecting neighboring grain vertices.

For the tested tools, the authors determined, among others, the following parameters: the area of the elevation above a specified plane, the coordinates of elevation vertices, the density of elevations, statistical parameters of vertex positions, and the development of the perimeter of the elevation base. Beneficial features of the tool surface include the stable renewable sharpness of grain vertices, and in precision machining also a significant number of micro-corners along a wide cutting front and low variation in the operating radii of cutting edges.

**Figure 18:**
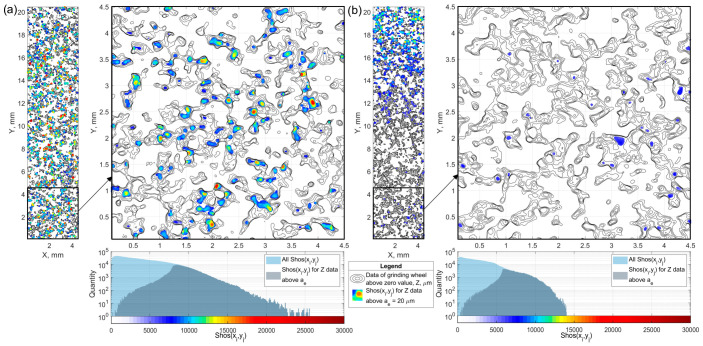
Local values of the *Shos* parameter for the grinding wheel A before the grinding process (**a**) before machining; (**b**) after 30 min of grinding.

**Figure 19:**
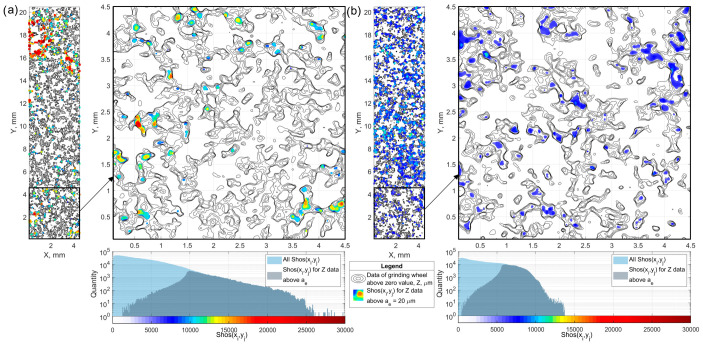
A histogram of local values of the *Shos* parameter for the grinding wheel B with a spatially developed grain structure: (**a**) before machining; (**b**) after 30 min of grinding.

**Figure 20:**
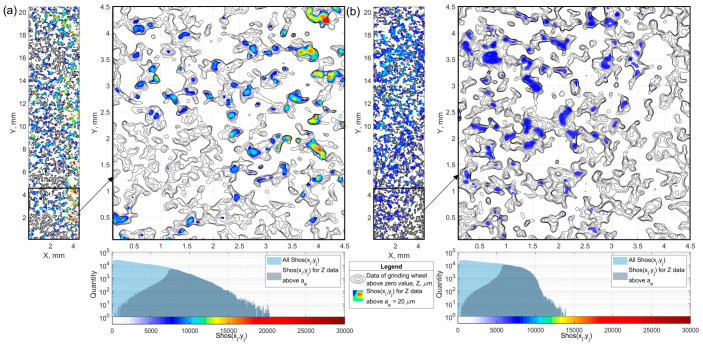
A histogram of local values of the *Shos* parameter for the grinding wheel C with a spatially developed grain structure: (**a**) before machining; (**b**) after 30 min of grinding.

To illustrate geometrical changes concerning active grains, Figure 18, Figure 19 and Figure 20 present changes in local values of the *Shos* parameter caused by wear. In the maps presented in this work, local values of *Shos*(*xi*,*yj*)*Shos*(*x_i*,*y_j*)*Shos*(*xi*,*yj*) were visualized only for the active surface regions located above the adopted infeed-related threshold level.

The comparison of local values and histograms of the *Shos* parameter before and after grinding makes it possible to assess changes in the state of active grain vertices. The decrease in the number of high and sharp vertices indicates blunting and wear of the active parts of the grinding wheel. Such information is important not only for the assessment of the current tool condition, but also for predicting the period in which the tool still has sufficient machining potential.

Spectral analysis was also applied to support the interpretation of changes in the grinding wheel active surface. The power spectral density (PSD) was determined from the measured surface height data as a function of the spatial component length in the X direction. This representation makes it possible to identify which spatial components of the wheel topography are most affected by grinding. In particular, changes in the PSD curve after grinding indicate modifications of the surface structure caused by wear, the blunting of active grain vertices, and the possible smoothing or clogging of inter-grain spaces. Therefore, PSD analysis should be treated as a complementary diagnostic tool to the *Shos* parameter: *Shos* describes the local machining potential of active vertices, whereas PSD describes changes in the spatial structure of the active surface at different length scales.

**Figure 21:**
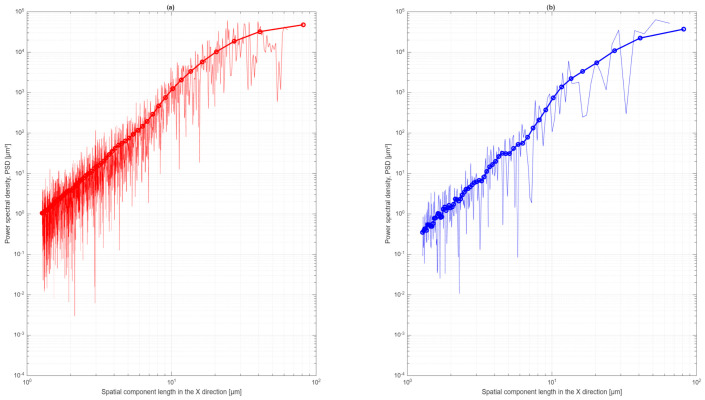
Power spectral density as a function of component length determined from grinding wheel A surface values: (**a**) before grinding; (**b**) after 30 min of grinding.

**Figure 22:**
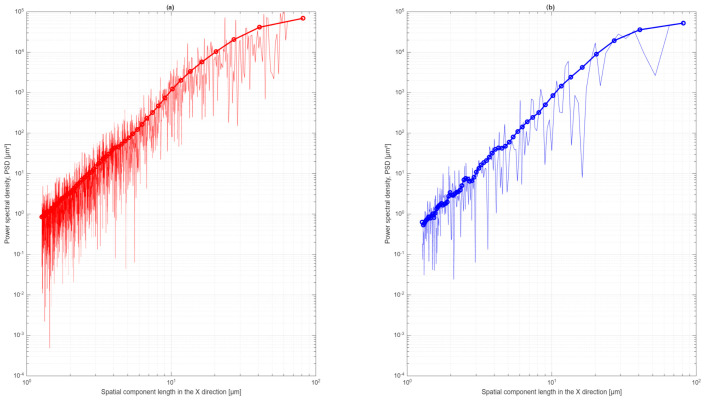
Power spectral density as a function of component length determined from grinding wheel B surface values: (**a**) before grinding; (**b**) after 30 min of grinding.

**Figure 23:**
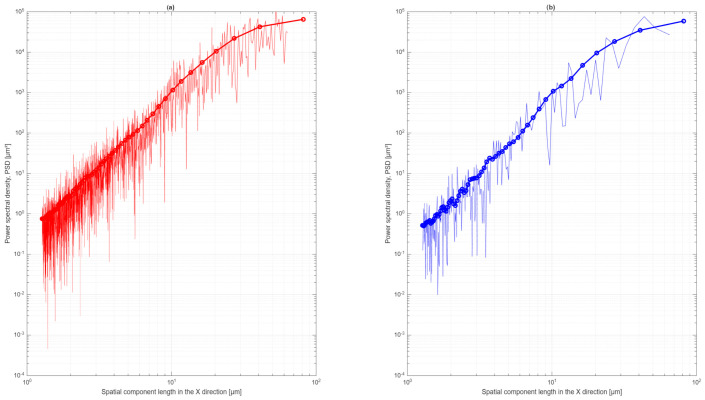
Power spectral density as a function of component length determined from grinding wheel C surface values: (**a**) before grinding; (**b**) after 30 min of grinding.

As can be seen from Figure 21, Figure 22 and Figure 23, an overall tendency of the PSD curves towards an increase in the value of their ordinates with increasing wavelength length can be observed for any of the studied grinding wheels. It means that the dominating part of the surface roughness measured is provided by large surface structures, i.e., surface protrusions of grains, pores, etc. Thus, in Figure 21, corresponding to grinding wheel A, the PSD curve prior to grinding reveals a rather wide variety of spectral components in terms of their wavelength distribution, as well as a large number of fluctuations in short- and medium-wavelength ranges, which testifies to the presence of many irregularities and sharp micro-structures on the surface of the grinding wheel. After grinding for 30 min, the PSD curve still grows; however, the contribution of fine- and medium-length spatial structures reduces, which reflects the process of rounding off the most sharp and protruded vertices of the active structures. For the grinding wheel B with increased porosity, represented in Figure 22, two PSD curves exhibit rather high growth in PSD values with increasing wavelength values, testifying to the dominating impact of large spatial structures, i.e., the porosity, on the overall surface roughness. After grinding, the resulting curve still contains rather significant fluctuations in medium- and long-wavelength ranges, testifying to the retention of the porous structure, even though some of the finest surface structures wear out. In Figure 23, representing grinding wheel C with spatially developed abrasive grains, the same tendency is demonstrated: after grinding, the PSD curve significantly decreases for fine and medium spatial components. However, for long spatial structures, the contribution still remains relatively high, which implies that the developed structure of grains still dominates on the grinding wheel surface. Thus, the comparative analysis performed above proves that grinding mainly impacts short- and medium-wavelength components related to active cutting vertices, whereas the contribution of long-wavelength components is more related to the structure of the grinding wheel surface.

The comparative analysis of the local *Shos* distributions and the corresponding power spectral density curves confirms that the tested grinding wheels differ not only in the number of active vertices, but also in the mechanism of active surface evolution during grinding. For the conventional grinding wheel A, high *Shos* values before grinding were distributed over numerous isolated active vertices, whereas after 30 min of grinding the number and intensity of high-value regions decreased markedly (Figure 18). This indicates blunting of the most protruding and sharp grain vertices and a reduction in the local machining potential of the active surface. In the case of grinding wheel B, characterized by increased structural porosity, the *Shos* maps show a more discontinuous distribution of active zones (Figure 19). This confirms that larger distances between active grains increase the openness of the active surface, but also concentrate the load on fewer active vertices. As a consequence, the wear process is reflected by a clear reduction in high *Shos* values after grinding. Grinding wheel C, containing spatially developed abrasive grains, showed a different behavior (Figure 20). Although the highest *Shos* values also decreased after grinding, numerous active regions remained visible. This suggests that the spatially developed grain structure may support the formation or exposure of new micro-cutting vertices during wear, which is consistent with the expected self-renewing action of aggregate-type abrasive structures. The PSD analysis provides complementary information to the *Shos* maps (Figure 21, Figure 22 and Figure 23). In all cases, the PSD curves increase with spatial component length, which means that the dominant contribution to the grinding wheel topography is related to larger spatial features associated with grain protrusions and the arrangement of active surface elements. After grinding, changes in the PSD curves are especially visible in the range of shorter and medium spatial component lengths. This range corresponds to fine surface features, such as sharp micro-vertices, local edges, and small-scale irregularities of abrasive grains. The decrease and redistribution of spectral components after grinding indicate the smoothing, blunting, and partial removal of the finest active features. Therefore, PSD analysis confirms the conclusions obtained from the *Shos* maps: grinding modifies the active surface mainly by reducing the intensity of the highest and sharpest vertices, while the remaining long-wavelength components describe the more stable spatial arrangement of grains and pores. The combined use of *Shos* and PSD is therefore justified, because *Shos* identifies local active cutting vertices and their wear, whereas PSD describes changes in the spatial structure of the grinding wheel active surface over different length scales. These results demonstrate that the conventional wheel, the wheel with increased structural porosity, and the wheel with spatially developed grains should not be interpreted as variants differing only in the number of active grains. They represent different mechanisms of active surface formation: stochastic protrusion of conventional grains, increased openness of the porous structure, and multi-vertex activity of spatially developed abrasive grains.

The methodology presented above makes it possible to assess those features of the grinding wheel surface that are important for the grinding process and for the development of abrasive tools. The state of the highest and sharpest grain vertices determines the ability of the tool to remove material, while changes in these features during machining describe the progress of wear. For this reason, parameters based on the analysis of the active surface, especially the *Shos* parameter, may be used in the diagnostics of abrasive tools, in the assessment of their machining potential, and in the development of new tool structures with improved durability and stability.

### 3.4. Material Removal by a Single Abrasive Grain and an Abrasive Aggregate

Simulation analysis was performed to describe the process of workpiece material removal in micro-cutting with a single abrasive grain and abrasive aggregate. Modeling material separation allowed the identification of the phenomena in the proximity of cutting edges of the grain and aggregate. The purpose of the work in this section of the research was to make a comparison between two types of abrasive morphology and show how the geometry of the aggregate can be applied in developing new abrasive tools.

#### 3.4.1. Material Flow in Micro-Cutting with a Single Grain

**Figure 24:**
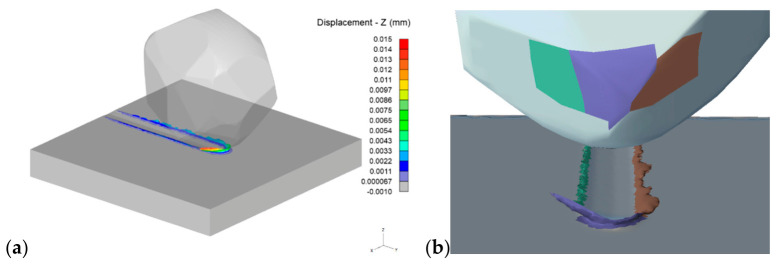
The results of the computer simulation of the cutting process with the abrasive grain: (**a**) material flow during cutting; (**b**) colored areas responsible for plastic flow of the workpiece material.

Based on the analysis of micro-cutting results, differences in pile-up and chip formation between the grain (Figure 24) and the aggregate can be observed. Geometrical parameters of the grain and aggregate affect the way in which the workpiece material flows.

**Figure 25:**
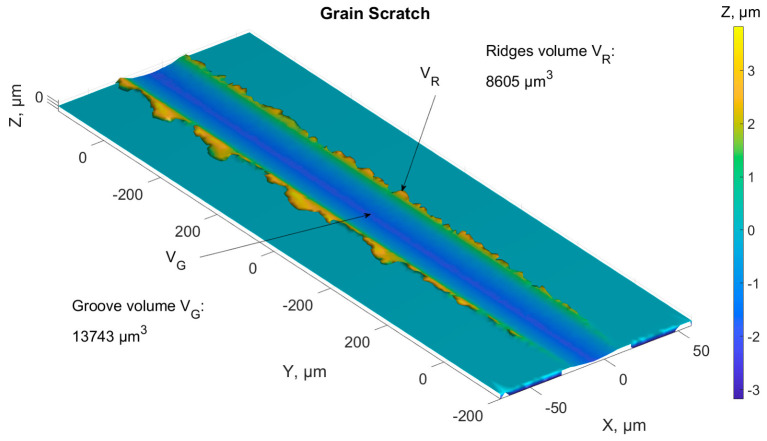
Scratch volume and lateral pile-ups after the grain cutting process for a depth of cut of 2 µm.

In micro-cutting with a single grain (Figure 25), the wedge-shaped grain geometry with cutting edge bz and two lateral edges b1 and b2 displaces material to the sides, forming a lateral chip. The area related to the longer lateral edge b2 displaces much more material than that related to the shorter lateral edge b1. The significantly negative rake angle γ = −78° at the penetration depth of 2 µm also favors material displacement to the sides.

As a result, this grain geometry causes a relatively small amount of the material volume to be removed as a chip, whereas most of the material volume is displaced to the sides in the form of lateral pile-ups. Scratch volume and pile-up volume after cutting with the abrasive grain confirm the above mechanism.

#### 3.4.2. Material Flow in Micro-Cutting with an Abrasive Aggregate

Wide cutting edge bz characterizes the abrasive aggregate geometry. The combined length of lateral edges b1 and b2 is nearly half the width of bz, providing lateral material-flow areas to be much smaller than those related to the cutting-edge area.

The rake angles of grains within the aggregate range from γ = −65° to γ = −73° at the penetration depth of 2 µm. In addition to a wide cutting edge, it allows the formation of wide chips. Thus, the workpiece material is not displaced to the sides but is removed from the cutting zone in front of the aggregate as a chip.

**Figure 26:**
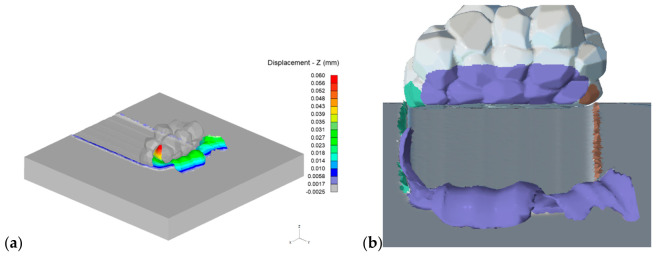
The results of the computer simulation of the cutting process with the abrasive aggregate: (**a**) material flow during cutting; (**b**) the colored areas are responsible for the plastic flow of the workpiece material.

**Figure 27:**
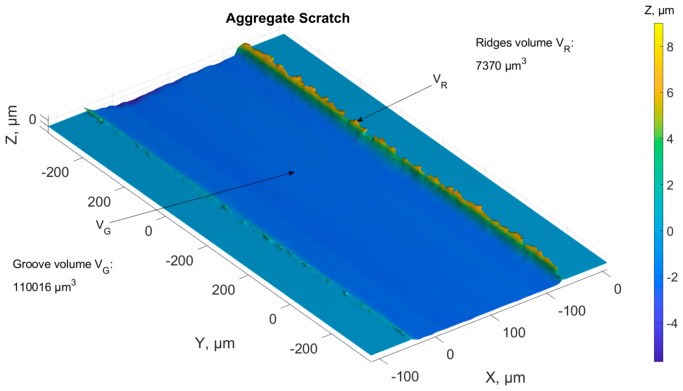
Scratch volume and lateral pile-ups after the aggregate cutting process for a depth of cut of 2 µm.

Scratch volume and lateral pile-ups after cutting with the abrasive aggregate (Figure 26) prove that the material volume removed as a chip is 15 times more than the pile-up volume (Figure 27).

The results of the simulations described in this section must be seen in relative context. They provide insight into the influence of abrasive geometry on the material removal mechanism under the assumed model conditions. Therefore, the obtained values of pile-up volume, chip formation, and material removal efficiency are used to compare a single abrasive grain with an abrasive aggregate, not to predict grinding forces, grinding temperature, tool wear, or surface quality in a real grinding process. The objective of the model was to isolate the geometrical effect of cutting-zone width and lateral material-flow regions on the material removal mechanism.

#### 3.4.3. Evaluation of Material Removal Efficiency

Previously, it was shown that geometrical parameters of abrasive grain cutting edges affect the way in which the material is displaced within the machining zone. The most significant parameters are the ratio of rake angle γ to apex angle ε of the abrasive grain, the ratio bb/bz of lateral displacement length to cutting-zone width, and the opening angle α of the abrasive grain.

Material removal efficiency for the grain and the abrasive aggregate was estimated using coefficient kr. This coefficient considers the volumes of lateral pile-ups on both sides of the cutting edge and the volume of material removed by the cutting edge. It is assumed that kr = 1 if all the material volume is removed as a chip and lateral pile-up volume is zero.

Analysis of the micro-cutting process shows that the material removal efficiency for cutting with the abrasive aggregate is much higher, specifically, kr = 0.93 for the aggregate and kr = 0.37 for the single grain. The above values show that aggregate geometry facilitates chip formation but limits pile-up formation.

Previously, it was found that the ratio bb/bz can be used to predict the material separation mechanism in micro-cutting. If bb/bz < 1, then material removal occurs as wide chips with small pile-ups; if bb/bz = 1, then material removal results in narrow chips with significant pile-ups; if bb/bz > 1, then chips with small volume are expected, mainly formed laterally, and pile-ups with large volume.

It should be noted that abrasive aggregates have a wide cutting edge bz and bb/bz ratio near or less than 1. Therefore, cutting-edge geometry allows the material to be mainly removed as chips. It is confirmed by high volumetric material removal efficiency.

The present case study indicates that abrasive particles are capable of forming an interesting trend in abrasive tool development. Under the assumed model conditions, the wider cutting front, smaller relative lateral-displacement regions, and more favorable rake geometry of the aggregate limited lateral pile-up formation and promoted chip formation. It should be stated that the results obtained do not constitute a complete validation of improved grinding performance in grinding under all machining conditions. However, they offer an understanding of the reasons behind better material removal. A wide cutting front, smaller lateral-displacement regions, and better rake angles allow the limitation of pile-up formation and the improvement of chip formation. As a result, abrasive aggregates can be considered not only as modifiers of conventional abrasive tools, but also as structural elements that change the mechanism of material removal in precision grinding.

## 4. Conclusions

- The development of abrasive tools should be considered not only from the point of view of abrasive material, bond type, or grinding parameters, but also from the point of view of the active surface structure. The number of active grains, their operating radii, the shape of grain vertices, and the possibility of renewing sharp cutting edges have a direct influence on the stability and efficiency of precision grinding. The issue takes particular significance when it comes to machining that has a narrow machining allowance, where only a small number of particles are involved in removing materials; the positioning of active vertices plays an important role here.
- The results obtained through the analysis performed in the present study show that the evaluation of the active surfaces of grinding wheels is an important part of designing and improving abrasives. The parameters obtained from the highest and the sharpest vertices of the grains, especially from the *Shos* parameter, help to detect any changes caused by wear and evaluate the performance capabilities of the tool. The additional use of the power spectral density (PSD) method helps to define the changes in the structure of the grinding wheel active surface at different length scales. As a result, using both methods, namely *Shos* and PSD, provides a more complete approach to diagnostics since *Shos* detects changes in the local active cutting vertices, whereas PSD detects changes in the structure of the whole active surface of the tool.
- The results also show that one of the most promising development directions is the use of abrasive aggregates and micro-aggregates. Their structure makes it possible to obtain a wider cutting front, a more favorable distribution of active micro-grains, and greater possibilities of shaping the porosity and properties of the grinding wheel. This gives more freedom in adapting abrasive tools to difficult machining tasks than in the case of conventional homogeneous tools.
- The analysis of material removal by simulation reveals that the aggregate geometry plays an important role under the given conditions in the micro-cutting modeling. In contrast to a single abrasive particle, the aggregate had a wider cutting region and a smaller ratio between the length of lateral shift and cutting area width. Accordingly, most of the material was removed during the simulation as chips, without any significant formation of lateral piles. The value of the material removal efficiency coefficient was much larger for the aggregate (kr = 0.93), compared to that for the single particle (kr = 0.37). This result can be regarded as the comparative evaluation of the material flow dynamics and not as a validation of grinding improvement. Further experimental work is necessary to quantify the effect of abrasive aggregates on grinding forces, temperature, surface integrity, tool wear, and process stability.
- The presented results indicate that abrasive aggregates should not be treated only as an additional modification of conventional grinding wheels. They may be considered as structural elements that change the mechanism of material removal in precision grinding. This is important for further development of abrasive tools intended for difficult-to-machine materials, small machining allowances, and processes requiring high stability of surface formation.
- Future work should focus on the experimental verification of different aggregate shapes, their share in the grinding wheel volume, and the properties of bonds used inside aggregates and in the main tool structure. It is also necessary to develop diagnostic methods that connect topography-based parameters, such as *Shos*, with the real wear state and useful life of abrasive tools.

## Data Availability

The original contributions presented in this study are included in the article. Further inquiries can be directed to the corresponding authors.
